# A rare case of empyema caused by septic arthritis of the sternoclavicular joint, successfully treated with surgical drainage

**DOI:** 10.1093/jscr/rjae359

**Published:** 2024-05-30

**Authors:** Sachie Koike, Masahisa Miyazawa, Nobutaka Kobayashi

**Affiliations:** Department of Thoracic Surgery, Japan Red Cross Society Nagano Hospital, 5-22-1, Wakasato, Nagano, Nagano 380-8582, Japan; Division of General Thoracic Surgery, Department of Surgery, Shinshu University School of Medicine, 3-1-1, Asahi, Matsumoto, Nagano, Japan; Department of Thoracic Surgery, Japan Red Cross Society Nagano Hospital, 5-22-1, Wakasato, Nagano, Nagano 380-8582, Japan; Department of Thoracic Surgery, Japan Red Cross Society Nagano Hospital, 5-22-1, Wakasato, Nagano, Nagano 380-8582, Japan

**Keywords:** empyema, septic arthritis of the sternoclavicular joint, surgical drainage

## Abstract

Septic arthritis of the sternoclavicular joint is a rare joint infection, and it sometimes leads to a chest wall abscess or mediastinitis. We report a case of a 70-year-old man who was diagnosed with empyema caused by an anterior chest wall abscess extended from septic arthritis of the sternoclavicular joint. It is very rare that arthritis causes empyema combined with an anterior chest wall abscess, and this is the first report of such a case. The patient was successfully treated with surgical drainage.

## Introduction

Septic arthritis of the sternoclavicular joint (SASCJ) is rare and accounts for ~0.5% to 1% of all joint infections [1]. It can sometimes lead to life-threatening complications, such as osteomyelitis, chest wall abscess, and mediastinitis [1]. We present a very rare case of SASCJ, which leads to empyema caused by an extended abscess of the anterior chest wall and is treated with surgical drainage.

## Case report

**Figure 1:**
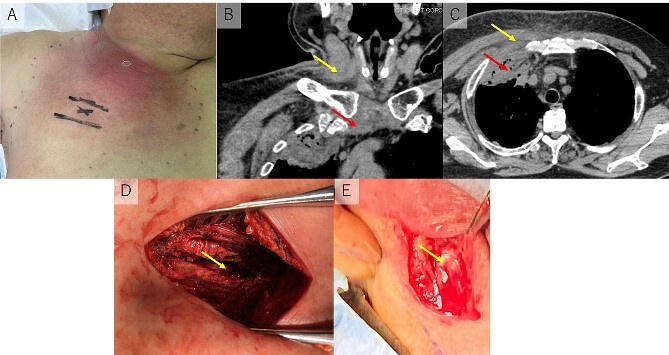
(A) Erythematous skin changes in the right anterior chest, neck, and shoulder. (B) The abscess was found in the right sternoclavicular joint (lower arrow) and right sternocleidomastoid muscle (upper arrow). (C) The abscess in the anterior chest wall (upper arrow) extended into intrathoracic space (lower arrow). (D) Surgical drainage was undergone for anterior chest wall and intrathoracic space (arrow). (E) The sternocleidomastoid muscle was split along the muscle fiber, and purulent collection was drained from the entire muscle (arrow).

A 70-year-old man consulted his primary care doctor due to severe pain in the right shoulder and chest wall that continued for 10 days. He had a history of diabetes mellitus (DM), asthma (treated with 5 mg/day of prednisolone), and hypertension. On physical examination, the patient was found to be febrile with a temperature of 38.6°C and tachycardic (140 beats per minute). His oxygen saturation in room air was 89%, and his blood pressure was 149/81 mmHg. He was noted to have swelling in the right anterior chest, neck, and shoulder. The swelling showed erythematous skin changes (Fig. 1A). Laboratory tests revealed an elevated white blood cell count (26.02 × 10^3^μl; normal range 3.3–8.6) and C-reactive protein level (32.51 mg/dl; normal range < 0.14 mg/dl). Chest computed tomography (CT) revealed abscess formation in the right sternoclavicular joint, right sternocleidomastoid muscle, and anterior chest wall. The abscess of the anterior chest wall extended into the intrathoracic space and developed an empyema (Fig. 1B and C). He was diagnosed with SASCJ combined with empyema and referred to our department. The patient was immediately started on 4.5 g of intravenous piperacillin/tazobactam (PIPC/TAZ).

On hospital Day 2, the surgical drainage was undergone with poor improvement of the patient’s clinical condition. First, we underwent drainage of empyema and anterior chest wall. A 10 cm incision was made on the midclavicular line of the second intercostal space, just above the empyema (Fig. 1D). An abscess was found in the interpectoral space, and the parietal pleura was impaired by strong inflammation of the intercostal space. From the intrathoracic space, a large amount of purulent collection was drained. Severe adhesion was made in the intrathoracic space. Second, the drainage of the abscess of right sternocleidomastoid muscle was undergone by otorhinolaryngologists. The sternocleidomastoid muscle was split along the muscle fiber, and purulent collections were drained from the entire muscle (Fig. 1E). Sternoclavicular joint space was not opened because the amount of the abscess in the space was small and no osteomyelitis was found on CT. A suction drain was placed in the thoracic space and the abscess cavity of the sternocleidomastoid muscle.

**Figure 2:**
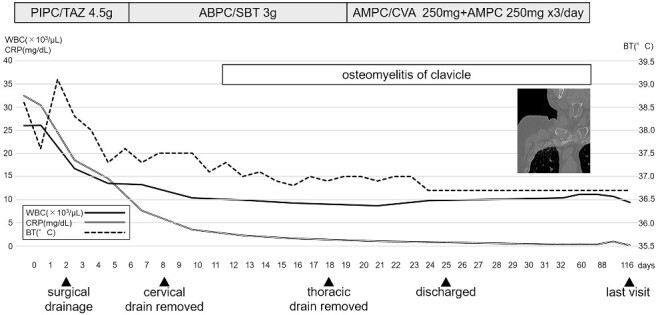
Clinical course. PIPC/TAZ piperacillin/tazobactam, ABPC/SBT ampicillin/sulbactam, AMPC/CVA + AMPC amoxicillin/clavulanate+ amoxicillin, WBC white blood cell count, CRP C-reactive protein level, BT body temperature.

Postoperatively, intravenous PIPC/TAZ was continued for 5 days, and after *Streptococcus agalactiae* was detected in the abscess cultures, the antibiotics switched to ampicillin/sulbactam. Although the postoperative CT on 11th hospital day revealed osteomyelitis of the clavicle, the patient’s general condition improved gradually, and inflammatory markers were trending down. The antibiotics switched to amoxicillin/clavulanate+ amoxicillin as oral medication, and the patient was discharged after 25 hospital days. The oral antibiotics were continued in an outpatient clinic for ~3 months, and the patient had no signs of recurrence until his last visit (Fig. 2).

## Discussion

SASCJ is mostly found in patients with underlying diseases such as diabetes, immunosuppression, and rheumatoid arthritis [1]. *Staphylococcus aureus*, *Streptococcus* species, *Escherichia coli*, and *Pseudomonas* are commonly reported causative agents [1].

SASCJ with pleural empyema is very rare, and only two cases other than ours have been reported in the literature [2, 3]. The relationship between SASCJ and empyema was reported as follows: (i) infection of SASCJ directly extended to the anterior pleura and caused empyema [2] and (ii) empyema necessitans led to SASCJ [3]. Different from these two cases, the empyema in our case is caused by an anterior chest wall abscess with the impairment of parietal pleura due to severe inflammation extended from SASCJ. This is the first report of such a relationship between empyema and SASCJ.

Glinski *et al.* [4] reported that en-bloc resection of the sternoclavicular joint is preferred in the definitive treatment of SASCJ, especially in cases of osteomyelitis, with a high recurrence rate (83%) of less-invasive approaches, such as antibiotics or surgical drainage. However, the patient in our case was successfully treated with limited surgery without joint resection. This case suggests that SASCJ can be successfully treated not only by definitive surgery but also with limited surgery like surgical drainage, especially in cases without osteomyelitis in the acute phase.

Currently, video-assisted thoracoscopic surgery (VATS) is widely used as an effective approach for empyema [5]. Although, in our case, we approached the empyema cavity by thoracotomy with a relatively small incision just above the cavity. We chose such an approach with the prediction of severe adhesion of the intrathoracic space, and as a result, this might be the least invasive and most effective approach in our case.

## Conclusion

In conclusion, we have reported a very rare case of SASCJ, which led to an empyema extending from the chest wall abscess. Surgical drainage should be one of the most effective treatments.

## Data Availability

The data underlying this article cannot be shared publicly for protecting privacy of individuals that participated in this study. The data may be shared on reasonable request to the corresponding author after an additional approval by the Institutional Review Board of Japanese Red Cross Society Nagano hospital, Japan.
